# A randomised controlled trial of the effects of a web-based PSA decision aid, *Prosdex*. Protocol

**DOI:** 10.1186/1471-2296-8-58

**Published:** 2007-10-04

**Authors:** Rhodri Evans, Glyn Elwyn, Adrian Edwards, Robert Newcombe, Paul Kinnersley, Pat Wright, Jeff Griffiths, Joan Austoker, Richard Grol

**Affiliations:** 1 Department of Primary Care and Public Health, Cardiff University, Wales, UK; 2 School of Psychology, Cardiff University, Wales, UK; 3 School of Mathematics, Cardiff University, Wales, UK; 4 Cancer Research UK Primary Care Education Research Group, Division of Public Health and Primary Health Care, University of Oxford, Oxford, UK; 5 Centre for Quality of Care Research (WOK), Radboud University, Nijmegen, Netherlands

## Abstract

**Background:**

Informed decision making is the theoretical basis in the UK for men's decisions about Prostate Specific Antigen (PSA) testing for prostate cancer testing. The aim of this study is to evaluate the effect of a web-based PSA decision-aid, *Prosdex*, on informed decision making in men. The objective is to assess the effect of *Prosdex *on six specific outcomes: (i) knowledge of PSA and prostate cancer-related issues – the principal outcome of the study; (ii) attitudes to testing; (iii) decision conflict; (iv) anxiety; (v) intention to undergo PSA testing; (vi) uptake of PSA testing. In addition, a mathematical simulation model of the effects of *Prosdex *will be developed.

**Methods:**

A randomised controlled trial with four groups: two intervention groups, one viewing *Prosdex *and the other receiving a paper version of the site; two control groups, the second controlling for the potential Hawthorn effect of the questionnaire used with the first control group. Men between the ages of 50 and 75, who have not previously had a PSA test, will be recruited from General Practitioners (GPs) in Wales, UK. The principal outcome, knowledge, and four other outcome measures – attitudes to testing, decision conflict, anxiety and intention to undergo testing – will be measured with an online questionnaire, used by men in three of the study groups. Six months later, PSA test uptake will be ascertained from GP records; the online questionnaire will then be repeated. These outcomes, and particularly PSA test uptake, will be used to develop a mathematical simulation model, specifically to consider the impact on health service resources.

**Trial registration:**

Current Controlled Trial: ISRCTN48473735.

## Background

*Prosdex *is a web-based decision aid to help men consider whether or not to have a Prostate Specific Antigen (PSA) test, potentially for prostate cancer [1]. It was developed in the context of the UK Prostate Cancer Risk Management Programme (PCRMP), a strategy, promoted by the UK National Cancer Screening Programme, which has, as one of its key goals, the promotion of informed decision making about PSA testing [2]. According to the strategy, men should only have a PSA test if they have received appropriate information and had the opportunity to make a decision – a decision which, for many, is difficult due to the uncertainty of prostate cancer testing. Despite its increasing incidence in men, the only widely-available test for prostate cancer, PSA, is limited not only by its poor sensitivity and specificity, but also by the uncertainty relating to the natural history and the management of the disease [2,3]. It is for these reasons that, unlike the USA, there is not a PSA screening programme in the UK. Moreover, the PCRMP strategy arguably reflects the tension between an evidence-based approach to population testing – PSA in this case – and the needs of individual men to make informed decisions about their own health.

Decision aids have been developed for a range of health conditions to facilitate informed decision making. Characteristically, the risks and benefits of different options are presented in a variety of formats and media, thereby helping patients in the process of values clarification, seen as fundamental for informed decision making. *Prosdex *was developed in 2002–04, supported by a grant from Cancer Research UK and the NHS Cancer Screening Programme [1]. Hosted by Cardiff University, with links from NHS Direct Online and Cancer Research UK, it presents evidence-based information about prostate cancer and PSA testing, encouraging users to weigh the pros and cons of testing for themselves. In addition, *Prosdex *includes video clips of enacted patient experiences about the PSA test and subsequent investigations/treatments. There is also information about 'shared decision making' and, through structured decision support (the 'decision stacker'), *Prosdex *aims to actively encourage informed decision making.

The aim of this proposed study is to evaluate the effect of *Prosdex *on informed decision making. In order to do so, a range of outcome measures need to be considered, due to the fact that a specific measure of informed decision making in PSA testing has not been developed. Three of the proposed outcome measures – knowledge, attitude to testing, and test uptake – are constituents of an informed decision making measure in another health context, prenatal Down syndrome [4]. These, and the other three proposed outcome measures – decision conflict, anxiety and intention to undergo testing – have been used in evaluations, specifically randomised controlled trials, of other PSA decision aids. In a systematic review of these trials, we found that knowledge increased by 19.5% and PSA testing decreased by 3.5% [5]. The six proposed outcome measures will, therefore, not only allow an assessment of the effect of *Prosdex *on informed decision making, but also will allow comparisons with other evaluations of PSA decision aids. This will hopefully enable an appraisal of implementation issues, for example the degree of awareness of the complexity surrounding PSA testing engendered in users by *Prosdex*[6]. Finally, a mathematical simulation model, using data from the trial, will allow extrapolations of the potential effects of *Prosdex *on health-service resource use, for example the impact on urological services, and on health outcomes.

## Methods

### i) Design

randomised controlled trial (RCT). This allows a comparison of the effect of a single intervention (*Prosdex*) on the specified outcomes in the objectives. The design employs four randomised groups of men in order to distinguish the effects of *Prosdex *from two other possible effects: format (electronic versus written), and the *Hawthorne *effect, specifically the effect that participating in a clinical trial could have on subsequent PSA uptake. RCTs have been used successfully to evaluate the effects of other PSA decision aids in North America [7,8].

### ii) Setting

Wales. Men recruited using GP lists.

### iii) Participants

#### a) Inclusion criteria

Men between 50 and 75 will be invited to participate, as prostate cancer is rare below the age of 50;[2] also, above the age of 75, men would be less likely, in our opinion, to complete the study, particularly the online questionnaire element. The men will access the study via the internet and must be able to use a computer. They will be asked to indicate this on the consent form. The numbers unable to participate due to this, in addition to those who fail to respond to the invitation, will be counted separately, in line with the CONSORT guidelines for reporting RCTs [9].

#### b) Exclusion criteria

Men who cannot read English will be excluded, as *Prosdex *was developed first only in English. Also excluded will be men who are known to have had prostate cancer and those whose GP records indicate that they have had a PSA test.

#### c) Recruitment process

Suitable men will be identified by GPs, in Wales, who will also send the invitation letters, participant information sheets (PIS) and consent forms. A member of the practice staff, probably the data manager, will be asked to identify men, aged 50–75, who have not had a PSA test. Using that generated list, the data manager will be asked to select 100 men using a serial recruitment process based on the date of the month of the men's birthdays: that is, the first man selected will be the first man on the list with a birthday 01/month/year; second man, 02/month/year; and so forth up to 31 when the process will be repeated until 100 men are selected. A member of the practice staff who has knowledge of the patients – Practice Manager or GP – will then be asked to screen the list for men who, in their opinion, are unsuitable for the trial due to serious ill-health. The number of men thus removed will be reported.

Affirmative consent forms from each practice will be transferred to the research officer who, in turn, will allocate each participant from that practice with a number provided the by trial statistician who will oversee the allocation process. Accordingly, the participants will be randomly allocated, by computer, to one of two intervention groups or to one of two control groups. This process will occur remotely in order to secure concealment. For each practice, the statistician will allocate 80 numbers, generated in 'blocks', the number for which will be between 12 and 16 in order to guarantee 'balance'. Randomisation will occur at the level of the man as we are interested in individual decision-making outcomes, and there is unlikely to be a significant intra-cluster correlation for these outcomes [10]. There will be no randomisation of the GP practices, but they will be stratified according to socio-economic groups, with an aim of 4 different such groups. After the collection of the data, there will be social analysis of these groups.

### iv) Intervention (see fig 1)

**Figure 1:**
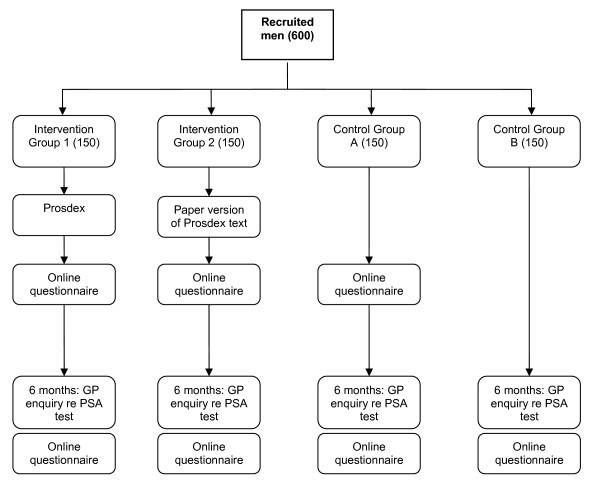
Intervention and control groups.

A specific version of the *Prosdex *website will be developed: it will require a password for access and will generate the online questionnaire. Men in intervention group 1 will be asked to log onto and view the website, either in their own homes or in another setting of their choice. The second intervention group (2) will receive a paper document comprising the text of the website. This enables evaluation of the Prosdex features (e.g. video clips and the structured decision support) that go beyond the mere presentation of the text content. In the first control group (A), men, after inserting their password, will be asked to complete the online questionnaire without viewing *Prosdex*. The second control group (B) will not initially be given the details of the study website.

There are, therefore, two main comparisons:

1) Intervention Group 1 v Control Group A:

*Prosdex *(+ online questionnaire) against no intervention, but with the online questionnaire: tests the effect of *Prosdex *content itself within the online context.

2) Intervention Group 1 v Intervention Group 2:

Two different formats to present almost identical content: tests the effects of the media: online versus paper-based.

The comparison between Control Group A and Control Group B allows a consideration of the *Hawthorne *effect of the questionnaire on PSA testing, to aid interpretation of outcomes in Intervention Groups 1 & 2.

At the six month stage, after the ascertainment of PSA testing status, men in the two intervention groups and control group A will be asked to repeat the online questionnaire. The purpose of repeating the questionnaire will be to evaluate any changes in the outcomes over time. Of particular interest is the effect on the principal outcome, knowledge, thereby allowing an assessment of knowledge retention. Men in control group B will also be asked at the six month stage to complete the online questionnaire in order to provide a control for the other three groups. All the men, therefore, at this six month stage, will be sent a letter asking them to access and complete the online questionnaire. This second questionnaire will have an additional question asking men to indicate, by 'left-clicking' on corresponding boxes, any types of information, newspapers/magazines for instance, they may have used in reaching a decision about how likely they are to have a PSA test.

### v) Outcomes

Six outcomes will be measured in this study: (a) knowledge of PSA and prostate cancer-related issues – this is the main outcome of the study, on which the sample size calculation is based; (b) attitudes to testing; (c) decision conflict; (d) anxiety; (e) intention to undergo PSA testing; (f) uptake of the PSA test. Outcomes (a) – (e) will be gathered from the online questionnaire. In addition to these outcomes, based on these results, a mathematical simulation model of the effects of *Prosdex *on subsequent resource use and health outcomes will also be developed. This model will be based on the results of the trial.

#### a) Knowledge of PSA and prostate cancer-related issues

Knowledge will be the principal outcome of this study. Previous randomised controlled trials of PSA decision aids have used knowledge as their principal outcome, and in our systematic review of these trials we found that PSA decision aids resulted in an improvement in knowledge of 19.5% [11]. Knowledge will be assessed using a set of knowledge questions, used in our earlier evaluation of a brief paper-based leaflet about PSA testing,[12] which showed an ability to discriminate between intervention and control groups.

#### b) Attitudes to testing

This will use a 12-item scale developed and used in the same evaluation of a brief paper-based leaflet about PSA testing [12].

#### c) Decision conflict [13]

This scale measures patients' confidence or uncertainty ('conflict') about whether they feel their choice is the best for them personally. It has acceptable validity and reliability (internal consistency alpha coefficients range from 0.78 – 0.89; test-retest reliability coefficients exceed 0.80) [14]. Given the nature of the decision about having a PSA test, with a high degree of uncertainty likely to affect decision making, it is important to use this, the most widely used outcome measure in decision aid studies [15].

#### d) Anxiety

This will be assessed using the short form Spielberger questionnaire for 'state' anxiety, validated and shown to be responsive in our earlier studies of shared decision making and risk communication [10].

#### e) Intention to undergo PSA testing

This will be assessed using a single item question, with Likert-like response scale, which has also been used in our earlier evaluation of a brief paper-based leaflet about PSA testing [12].

#### f) Uptake of the PSA test

This will be assessed at six months after the intervention. GPs who participate in the study will be asked to ascertain the men's PSA testing status for that six month period, from their records, and inform the research team whether or not the test was done. The GPs will be provided with specific forms for this purpose, to be returned to the research team. It is possible that men may have had PSA tests elsewhere, such as via hospital clinics, but it is likely that these will be evenly distributed across the intervention and control groups. Moreover, it may be less likely that these decisions to be tested were patient-led. Such 'external' results, when they do occur, are increasingly recorded in GP records.

### (vi) Comparisons and analysis

Comparability of the four groups for baseline characteristics of age, ethnicity, marital status and education will be assessed. Outcomes will be compared between groups on an 'intention to treat' basis by standard statistical tests, including chi^2 ^for categorical variables and t, Mann-Whitney and one-way ANOVA tests for continuous and ordinal variables. Point and interval estimates for appropriate measures of effect size will be reported as well as p values. A clinically significant and relevant difference in the principal outcome, knowledge, between the two groups, will be set at 20%. The statistical power for this study is aimed at 90%, assuming a type 1 error rate of 5%. The sample size will be 600 men: 150 in each of the four groups. This figure is derived from the findings of our systematic review of PSA decision aids where 4 RCTs (USA) were found to result in improved knowledge of 19.5% (SD 45.1). Thus, 150 men per group will allow the detection of a 20% absolute difference with over 90% power. Assuming a recruitment and completion rate of 30%, 2000 men will be invited from 20 GP practices, 100 men from each practice. For the Decisional Conflict Scale, a comparison of any 2 groups each of 150 subjects would detect a shift of 0.32 standard deviations, with power 80% at the conventional 5% alpha level. The data will be collected in a SQL-server database, transferred to Excel, and then analysed using SPSS Syntax, and the results expressed with both p values and confidence intervals.

### vii) Mathematical Simulation Model

A mathematical simulation model of the effects of *Prosdex *on subsequent resource use and health outcomes will also be developed. This will use the trial outcomes, particularly PSA uptake, to model potential diagnoses and morbidity (drawing on existing best evidence [16]) and the resulting resource use and workload implications at GP practice and NHS Trust levels. For example, at GP practice level, the age distribution in a practice will be used, together with PSA take-up after the intervention, to estimate workloads and resource requirements, using appropriate sampling distributions as in the investigator's previous research.[17,18] By further sampling of GP practices within a hospital Trust locality, estimates would be made of the implications at Trust level for urological and other services. The data will be obtained for local GP practices from our network of practices who have participated in several other research studies in recent years.

## Abbreviations

PSA: Prostate Specific Antigen; GP: General Practitioner; PCRMP: Prostate Cancer Risk Management Programme; RCT: randomised controlled trial; CONSORT: consolidated standards of reporting trials.

## Competing interests

The author(s) declare that they have no competing interests.

## Authors' contributions

All authors contributed to the design of this study. RE is responsible for the management of the trial, and GE is the principal investigator. All authors read and approved the final manuscript.

## Pre-publication history

The pre-publication history for this paper can be accessed here:


